# The relationship between clinical measures of cognitive function and grip strength in healthy older adults

**DOI:** 10.1186/s12877-022-03629-9

**Published:** 2022-11-26

**Authors:** James K. Richardson, Toby J. Ellmers

**Affiliations:** 1 Department of Physical Medicine and Rehabilitation, University of Michigan, 325 E. Eisenhower Pkwy, Suite 200, Ann Arbor, MI 48108 USA; 2 Department of Brain Sciences, Imperial College of London, London, UK

**Keywords:** Grip strength, Cognition, Executive function, Reaction time

## Abstract

**Background:**

Handgrip strength is considered a surrogate for musculoskeletal strength, however there is emerging evidence of an association with cognition. The specific neurocognitive attribute which best associates with grip strength is unknown.

**Methods:**

We performed a secondary analysis on baseline data in 49 healthy older adults. Grip strength was corrected for body mass index. Control independent variables included age, Montreal Cognitive Assessment, and Trails B. Experimental variables included a clinical measure of simple reaction time, and clinical and computerized go/no-go tasks. The clinical Go/No-Go measure was determined with ReacStick, a rod-shaped device which – when released by the examiner – requires the participant to decide within 390 ms whether to catch the device or let it fall to the ground.

**Results:**

Bivariate analysis demonstrated that age and all cognitive measures other than the computer go/no-go response accuracy related to grip strength. Multivariate analyses showed that following inclusion of the control variables, only ReacStick measures (reaction accuracy/simple reaction time) significantly predicted grip strength, explaining an additional 15.90% variance (*p* = 0.026). In contrast, computerized Go/No-Go accuracy (*p* = 0.391), response time variability (*p* = 0.463), and the control variables (*p* value range = 0.566–0.942) did not predict grip strength.

**Conclusion:**

A short latency (< 390 ms) visuomotor Go/No-Go task independently predicted over 15% of grip strength variance, whereas a slower screen-based Go/No-Go task did not. These findings support the notion that declining grip strength likely reflects sub-clinical brain changes as well as musculoskeletal dysfunction, possibly explaining the potent relationships between grip strength, disability, chronic disease, and mortality.

## Background

Handgrip strength is a convenient clinical attribute which predicts functional limitations and physical frailty [1, 2]. Although handgrip strength is often considered a surrogate for overall musculoskeletal strength, a recent review offers evidence of an association between grip strength and general cognitive function (e.g. Mini-Mental Status Examination scores) [3]. Grip strength has also been found to predict longitudinal alterations in various measures of cognitive function [4–7], including predicting changes in cognitive performance up to 9 years later [8]. In further support of a cognition-grip strength relationship, brain imaging findings have also been associated with grip strength. For example, diminished grip strength has been related to increased number of cerebral white matter hyperintensities [5, 9], whereas greater grip strength is associated with increased brain grey matter volume [8]. Other research in older people with and without major depressive disorder found that grip strength was predicted by hippocampal volume as well as white matter hyperintensities [10]. Finally, research on dizygotic and monozygotic twins suggests that common genetic factors predict grip strength, cognitive processing speed and working memory [11]. Taken together, these findings suggest clear links between grip strength and cognitive function.

Emerging evidence suggests that intact inhibitory executive function is required for optimal muscle force production. For example, compared to young adults, middle-aged people demonstrated diminished inhibitory function and grip strength performance, with these significantly related [12]. Further, young people instructed to maintain a steady force output when distracted with an auditory stimulus required inhibitory cortical activity about 300 ms after stimulus presentation in order to do so [13]. These findings suggest that inhibitory executive function may be particularly important for exerting optimal as well as maximal muscle force. Despite this, the relationship between cognitive inhibition and grip strength in older adults has not been fully explored. We hypothesize that the association between grip strength and cognitive inhibition would be stronger than that between grip strength and measures of generalized global cognitive or executive function.

Using previously obtained data in healthy older people [14], we performed a secondary analysis evaluating the relationship between cognitive measures and grip strength. More specifically, we evaluated the relationships between grip strength and measures of general intellectual and executive function, in addition to computerized and clinical measures of inhibition and cognitive processing speed. The latter was evaluated by ReacStick, a novel, validated measure of short latency cognitive inhibitory function which requires a go/no-go response within 390 ms [15, 16]. We hypothesized that there would be relationships between grip strength and all the cognitive measures, but that ReacStick would show the strongest association due to its requirement for inhibitory function and cognitive processing speed.

## Methods

### Overview

The herein reports a secondary analysis on previously collected baseline assessment data [14]. Data is presented for 49 healthy community-dwelling older adults recruited from local community groups and without known neurologic, vestibular, or musculoskeletal disease or disorder, and no participant reported any upper limb pain. The last was confirmed prior to administering grip strength testing so as to avoid injury. Institutional ethical approval was obtained and the research performed in accordance with principles laid down by the Declaration of Helsinki. All participants provided written informed consent prior to participation.

### Dependent variable

Grip strength was assessed using an electronic hand dynamometer (Camry, CA, USA). Participants sat with their elbow flexed at 110°. Participants squeezed the dynamometer handle as hard as they could three times (separated by 30 s rest) with their dominant hand, and then repeated the protocol with their non-dominant hand [17]. The highest value for each hand was recorded and the mean of these two values, divided by participant body mass index (BMI), served as the dependent variable.

### Independent variables

#### Cognitive testing

The Montreal Cognitive Assessment (MoCA) and Trails-B test were administered in accordance with established recommendations given the status of these evaluations as well-validated measures of general cognitive and executive functions, respectively [18, 19].

#### ReacStick

**Fig. 1:**
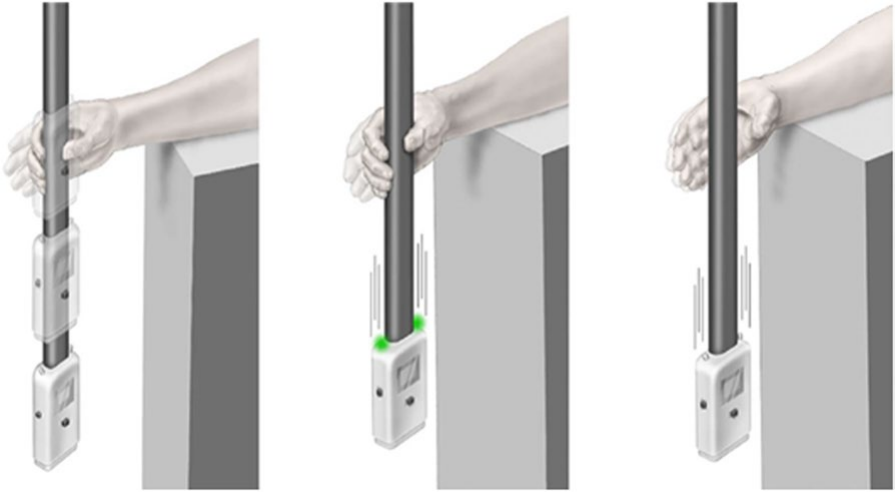
ReacStick as used to determine simple reaction time (SRT) and reaction accuracy (RA). The panel on the left depicts measurement of SRT which is measured initially. To evaluate SRT the device is dropped from desk height (74 cm) by the examiner at random intervals and the participant catches it as quickly as possible. RA is depicted in the middle and right sided panels. To evaluate RA the device mode is changed so that the green lights affixed to the top of the spacer box illuminate randomly 50% of the time at the instant of release. The participant is instructed to catch the device only on the trials in which the lights illuminate (middle panel) and to let the device strike the floor when the lights do not illuminate (right side panel). Following SRT there is a pre-potent response to catch the device, and so successful RA requires that this response be inhibited, light illumination status evaluated, working memory accessed, and action taken (or inhibited) within a 390-ms interval. The interval is similar to that allowed for return of a professional tennis serve (470 ms) [23] and clinically relevant in that movement must be initiated within 350 ms to avoid hip fracture during a direct lateral fall [24]

The ReacStick apparatus and procedure have been described and validated in detail elsewhere [15, 16, 20]. In brief, the apparatus is a lightweight rod affixed to a spacer box containing an accelerometer, microprocessor, light emitting diodes, and a display which provides the elapsed time between initial acceleration and deceleration (Fig. 1, total weight approximately 450 g). Importantly, ReacStick need not be grasped or arrested to trigger its time output or register a “catch” because the high friction handle allows activation of the sensitive accelerometer with light touch or grazing by the hand/fingers, without need to arrest the falling device. This feature allows evaluation of people with altered grasp strength and hand dexterity. Simple reaction time (SRT) is determined by the examiner releasing the device at random intervals after its suspension between the participant’s dominant hand fingers which are held 1–2 cm from the device spacer box (Fig. 1). Four practice trials are followed by 8 data acquisition trials. To evaluate response accuracy (RA), ReacStick mode is changed such that lights illuminate upon release during a random 50% of the trials. Participants are instructed to catch the device when the lights illuminate and to let it drop when they do not. Verbal instructions emphasized accuracy rather than speed. Released from desk height (74 cm) RA requires the participant to overcome the pre-potent urge to catch the device, access working memory, and execute a plan within a 390-ms interval. Participants are allowed 6 practice trials followed by 20 data acquisition trials. The percentage of appropriately caught/let drop trials is used for analyses. We selected RA:SRT as the most salient prediction variable because it rewards accuracy and speed, and best predicted motor functions in prior research [21, 22]. Test re-test reliability for SRT and RA are > 0.90 and 0.70, respectively [16]. Biomechanical analyses demonstrated that the majority of variation in SRT is related to pre-motor time, confirming that SRT is an evaluation of attention and processing speed rather than digit motor function [15]. RA evaluates executive functions, including decision-making, inhibition, selective attention, and working memory, as well as processing speed.

#### Computer Go/No-Go task

Following the ReacStick protocol, participants completed a computerized Go/No-Go task to assess inhibition (programmed on the open-source software PsyToolkit [25]). Every two seconds, an oval appeared on the screen. This oval was either green and displayed the word “Go”, or red and displayed the words “No-Go”. Participants were instructed to press the spacebar as fast as possible during Go trials (response activation), and to do nothing during the No-Go trials (response inhibition). The task included ten practice trials with feedback, and one test block with 100 trials without feedback (Go/No-Go ratio 4:1) [26]. As incorrect responses during No-Go trials are argued to best reflect inhibition (whereas incorrect responses during Go trials are argued to best reflect inattention) [25], this was selected as the most salient accuracy variable. Whilst 51% (25/49) of participants had some form of No-Go error, most of those participants (19/25) made only a single error. As such, this outcome was treated as a dichotomous (error present: Yes/No), rather than continuous, variable. Given that response time variability during Go trials correlates with neural activity related to inhibitory function [27, 28], we also calculated this as a secondary outcome. To control for differences in mean response time, we calculated response time variability using intra-individual coefficient of variation (ICV = Go response time SD/Go response time mean [29]).

### Statistical analyses

Grip strength and all cognitive variables except computer Go/No-Go Accuracy were normally distributed, and so relationships between these cognitive measures and grip strength were evaluated with Pearson corelations coefficients. Data were then analyzed using a linear regression, with mean grip strength/BMI the dependent variable. Control variables were entered in the first step. These were: age, general cognitive function (MoCA) and executive function (Trails-B). Predictor inhibition variables were added into the second step. These were: RA:SRT, computerized Go/No-Go accuracy and computerized Go/No-Go response time variability. The assumptions of homoscedasticity (inspecting the standardized residuals by standardized predicted values plot), error-independence (Durbin–Watson value = 2.31), lack of multicollinearity (variance inflation factors < 1.9, tolerances > 0.5), and normal distribution of errors (determined with Kolmogorov–Smirnov tests and inspection of histogram of residuals) were verified.

## Results

Participant mean age was 74.4 ± 7.2 years and thirty-four (69.3%) of the participants were female. Demographic and cognitive variable means, standard deviations, and correlations with grip strength are shown in Table 1. As reported in Table 1, bivariate analyses found that grip strength showed significant relationships with age and all cognitive measures. The cognitive measures with the strongest relationships with grip strength were RA and RA/SRT.

**Table 1:** Demographic variables and their relationships with grip strength. Note that computerized Go/No-Go Accuracy is not included as the data were not normally distributed. This variable was treated as a dichotomous outcome in the multivariate analyses

Participant (*n* = 49) Demographic and Cognitive Variables	Mean ± Standard Deviation	Correlation (*r*-/*p*-value) with grip strength/BMI
Age (yrs)	74.4 ± 7.2	**-0.353/0.012**
BMI (Body Mass Index)	25.9 ± 3.9	
Number of Medications	2.7 ± 2.3	-0.144/0.324
Grip Strength/BMI (kg/BMI)	2.12 ± 0.85	
Montreal Cog Assessment	26.5 ± 2.8	**0.290/0.041**
Trails B (sec)	93.6 ± 46.9	**-0.378/0.007**
Computerized Go/No-Go Accuracy (%)	96.3 ± 5.4	-0.188/0.191
Computerized Go/No-Go RT Variability (msec)	0.25 ± 0.08	**-0.298/0.036**
ReacStick SRT (msec)	177.5 ± 15.32	**-0.343/0.015**
ReacStick Accuracy (%)	68.6 ± 16.0	**0.450/0.001**
ReacStick Accuracy/SRT (%/msec)	0.39 ± 0.098	**0.547/ < 0.001**

Multivariate analyses demonstrated that introducing the three inhibition variables (RA/SRT, computerized Go/No-Go accuracy and response time variability) to the model containing control variables significantly improved model fit, explaining an additional 15.90% variance (*p* = 0.026). However, the only significant independent predictor was the RA:SRT (*p* = 0.013). In contrast, neither computerized Go/No-Go accuracy (*p* = 0.391) nor response time variability (*p* = 0.463), nor any of the control variables (*p*-values between 0.566–0.942), significantly predicted grip strength (Table 2).

**Table 2:** Results of linear regression analyses

**Dependent variable:** Grip strength (normalized to body mass index)					
	*B* (*SE*)	[95% CI]	*p*	*R*^2^	*R*^2^ change
**Step 1**				.190 (***p* = .023**)	
Constant	3.24 (1.98)	[-.755, 7.23]	.109		
Age	-.025 (.019)	[-.064, .014]	.203		
Cognitive function (MoCA)	.041 (.048)	[-.057, .138]	.404		
Executive function (Trails-B)	-.004 (.003)	[-.010, .003]	.250		
**Step 2**				.348 (***p* = .005**)	.159 (***p* = .026**)
Constant	2.01 (2.15)	[-2.38, 6.35]	.355		
Age	-.011 (.019)	[-.050, .028]	.566		
Cognitive function (MoCA)	-.003 (.048)	[-.099, .093]	.942		
Executive function (Trails-B)	-.001 (.003)	[-.008, .005]	.696		
ReacStick accuracy:response time	3.95 (1.52)	[.896, 7.01]	**.013**		
Go/No-Go accuracy	-.192 (.221)	[-.639, .255]	.391		
Go/No-Go response time variability	- 1.24 (1.68)	[-4.63, 2.14]	.463		

## Discussion

Consistent with other research [4–6], grip strength in our cohort was significantly and inversely related to age and directly related to accepted measures of general cognitive and executive functions. Multivariate techniques revealed that after accounting for these accepted measures of cognition, RA/SRT which simultaneously assesses both cognitive inhibition and processing speed independently explained over 15% of grip strength variance in a healthy older population. Of interest, age, accepted measures of general cognitive and executive functions, and a computerized measure of inhibition did not predict grip strength when this short latency reaction task was included concurrently. Given that grip strength has been accepted as a surrogate for overall musculoskeletal strength [30, 31], our findings are relevant in the research and clinical domains. The data suggest that clinical measures of musculoskeletal strength are not only measures of neuromuscular and musculoskeletal integrity, but also indicators of neurocognitive function. This point is buttressed anatomically by recent reports linking grip strength and changes in brain imaging [5, 9, 10]. Accordingly, researchers and clinicians need to account for cognitive function when evaluating strength, and the clinicians should consider interventions which may improve cognition (e.g., sleep disorder or polypharmacy) when treating functionally relevant weakness.

The finding that the ReacStick measure combining processing speed and cognitive inhibition (RA:SRT) predicted grip strength, whereas a computerized Go/No-Go task did not, requires exploration as both appear to require similar cortical resources (given that both contain a Go/No-Go component). Aron et al. [32] presents convincing evidence that rapid inhibition requires frontal cortical function (right inferior and/or supplementary motor areas) which communicates via a “hyper-direct” white matter tract with the sub-thalamic nucleus, which in turn excites the globus pallidus leading to inhibition of the basal ganglia. Following this inhibition, intact working memory is needed to select the correct response for “Go” vs. “No-Go” stimuli. This is followed by continued inhibition for a No-Go stimulus; or in the case of a Go stimulus the premotor cortex excites the basal ganglia, leading to the desired movement. Each of these steps should be required for accurate ReacStick and Computer Go/No-Go responses. The key difference is that in the case of ReacStick the response must occur within 390 ms, whereas the Computer Go/No-Go responses may take longer (given that participants have up to 2000 ms to respond). Accordingly, the mean latency for ReacStick Go trials was 259 ± 31 ms vs. 502 ± 85 ms for successful computer Go responses. Thus, ReacStick places a premium on response speed. Considering this model of inhibition, we hypothesize that accurate ReacStick response requires optimal integrity of the cortical to sub-thalamic nucleus hyper-direct white matter tract, whereas the integrity of this tract is of lesser relevance for the slower computer Go/No-Go responses. Therefore, ReacStick Accuracy may reflect white matter integrity as well as cortical resources, whereas the Computer Go/No-Go task is primarily evaluating cortical integrity. In support of this, other studies have found relationships between grip strength and both white matter integrity and reaction time [7, 9, 10, 33].

The key limitation of the present work relates to the relatively small sample size studied. Our study results must therefore be considered preliminary. Further, even these findings cannot be extrapolated to groups other than healthy, community dwelling older adults. Additionally, co-morbidities and specific medication use were not accounted for and may have introduced unaccounted for bias.

In summary, we found that RA:SRT performance which represents a short latency visuomotor Go/No-Go task combined with reaction speed independently predicted over 15% of grip strength variance, whereas a computerized Go/No-Go task and accepted measures of general cognitive and executive functions did not. Given that ReacStick accuracy requires a response within 390 ms, and central neurologic speed is mediated by white matter tract integrity, we tentatively suggest that ReacStick accuracy is dependent on white matter tract integrity along with the cortical resources needed for accurate Go/No-Go responses. If so, then declining grip strength likely reflects the presence of sub-clinical brain changes as well as more peripheral neuromuscular function. This possibly helps explain the potent relationship between grip strength, disability, chronic disease and premature mortality [34, 35].

## Data Availability

The datasets used and/or analyzed during the current study available from the corresponding author on reasonable request.
